# Molecular mechanisms of etoposide

**DOI:** 10.17179/excli2015-561

**Published:** 2015-01-19

**Authors:** Alessandra Montecucco, Francesca Zanetta, Giuseppe Biamonti

**Affiliations:** 1 Istituto di Genetica Molecolare, CNR, via Abbiategrasso 207, Pavia; 2 Dipartimento di Biologia e Biotecnologia, Università degli Studi di Pavia, via Ferrata 9, Pavia, Italy

## Abstract

Etoposide derives from podophyllotoxin, a toxin found in the American Mayapple. It was first synthesized in 1966 and approved for cancer therapy in 1983 by the U.S. Food and Drug Administration (Hande, 1998[25]). Starting from 1980s several studies demonstrated that etoposide targets DNA topoisomerase II activities thus leading to the production of DNA breaks and eliciting a response that affects several aspects of cell metabolisms. In this review we will focus on molecular mechanisms that account for the biological effect of etoposide.

## Etoposide is a Topoisomerase II Poison

### Topoisomerase II-mediated DNA breaks

**Figure 1:**
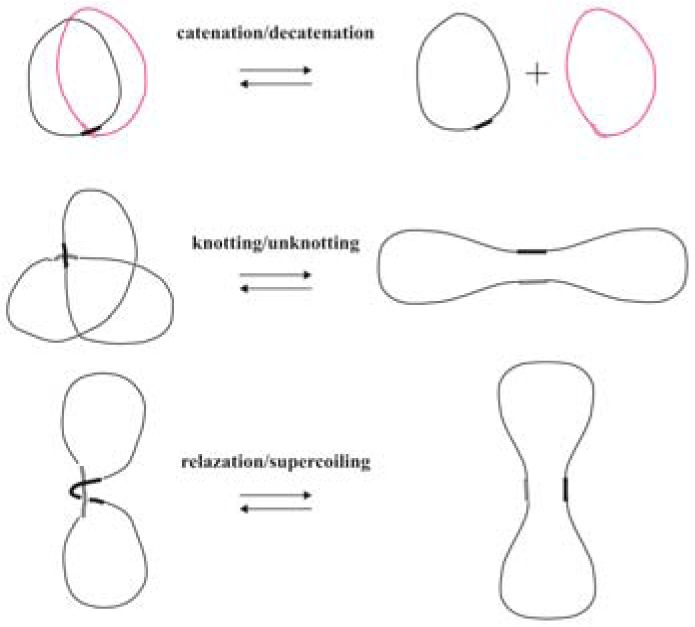
Reactions catalyzed by type II topoisomerases. All these transformations are performed by passing one double-stranded DNA segment through another.

DNA topoisomerases are essential enzymes that regulate the topological state of the genetic material by introducing transient breaks in the DNA molecule. They are involved in fundamental biological processes such as DNA replication, transcription, DNA repair and chromatin remodeling. The unwinding and rewinding of the double helix, the protein movement along DNA and the coiling of DNA in higher-order structures lead to topological entanglements that are resolved by topoisomerases by enabling topological transformation through two transesterification reactions. A tyrosine in the active site of the enzyme initiates the first transesterification and forms a covalent adduct with the phosphate in the backbone of the DNA molecule, thus generating a transient interruption of the double helix through which topological transformations can occur. The second transesterification reseals the DNA break (re-ligation) and regenerates the free tyrosine. Based on their structure and mechanism of action, topoisomerases are grouped into type I (TopoI) and type II (TopoII) enzymes. TopoI enzymes carry out strand passage through a single-stranded break, while TopoII activities involve the production of a double-strand break (Chen et al., 2013[11]). TopoII can perform three kinds of reactions (Figure 1(Fig. 1)): while DNA relaxation is in common to TopoI, catenation/decatenation and knotting/unknotting are TopoII specific. 

The covalent topoisomerase-cleaved DNA complex, referred to as cleavable or cleavage complex, is a short-live intermediate in this reaction. However, it can be stabilized by several compounds leading to the production of high levels of protein-associated breaks in the genome that are extremely toxic for the cell. 

Etoposide poisons the TopoII cleavage complexes (TopoIIcc) and inhibits the second step of the reaction (i.e. DNA re-ligation). The recent high-resolution of the ternary complex between TopoII, DNA and etoposide has revealed the elements that are crucial to the stabilization of the cleavable complex. Interactions with specific amino acids of the enzyme are critical for etoposide to enter the TopoII-DNA complex. The active role played by TopoII in promoting and stabilizing the ternary complex is consistent with the notion that the drug by itself displays low-affinity toward free DNA and is a poor DNA intercalator (Wu et al., 2011[80]; Wilstermann et al., 2007[78]). However a recent analysis indicates that etoposide has also a high-affinity for chromatin and histones, in particular H1, suggesting that beside TopoII, chromatin can be a target of the drug (Chamani et al., 2014[9]).

### Clinical implications 

Mammals have two TopoII isoenzymes, TopoIIα and β that are differently regulated during cell growth (Nitiss, 2009[56]). TopoIIα is a proliferation marker and is greatly elevated in tumor cells, whereas the β isoenzyme is present in proliferating as well as post-mitotic cells. In agreement with this differential expression TopoIIα functions in cell cycle events such as DNA replication and chromosome segregation, while TopoIIβ has been implicated in transcription and is associated with developmental and differentiation programs (Yang et al., 2000[82]; Lyu et al., 2006[43]; Tiwari et al., 2012[72]). Although both TopoII isoenzymes are targets of etoposide, the relative contributions of TopoIIα and TopoIIβ to the chemotherapeutic effects has yet to be resolved. Because TopoIIα is overexpressed in tumor cells, it is an ideal target for anticancer drugs. However, it is still unclear whether the two isoenzymes play different roles in tumor-cell killing in response to etoposide or more in general to TopoII-based chemotherapy. 

The issue of isoform specificity has potential clinical implications. For instance, since TopoIIα is not expressed appreciably in quiescent cells, etoposide targeting of TopoIIβ in differentiated tissues, such as cardiac cells, could account for much of the off-target toxicity of the drug (Azarova et al., 2007[3]). TopoII activity can be also involved in the drug-induced secondary malignancies, such as acute myelocytic leukemia (t-AML) and treatment-related myelodysplastic syndromes (t-MDS) often progressing to t-AML (Pedersen-Bjergaard et al., 2002[60][61]), that have been noted in patients receiving TopoII-based chemotherapy. 

Etoposide-induced t-AML is frequently associated with balanced translocations between the mixed lineage leukemia (*MLL*) gene on chromosome 11q23 and several partner genes. This translocation appears to be linked to the trapping of a TopoIIcc in the *MLL* gene (Lovett et al., 2001[41][42]). In fact the break point cluster region in the *MLL *gene is AT-rich and contains *Alu *sequences, corresponding to putative recognition sites of TopoII-mediated DNA cleavage, and chromosome scaffold/matrix attachment regions (SAR/MAR). 

Azarova and colleagues (Azarova et al., 2007[3]) used skin-specific TopoIIβ-knockout mice to test whether the two isozymes have different roles in the development of secondary malignancies and in tumor-cell killing. Their results suggest that, at least in this experimental system, TopoIIβ is the isoform responsible for etoposide-induced carcinogenesis. Furthermore, in cell-culture models, etoposide-induced DNA sequence rearrangements and DSBs are also found to be primarily TopoIIβ-dependent. By contrast, etoposide cytotoxicity in tumor cells expressing both isoenzymes appears to be mainly TopoIIα-dependent. Therefore, the two isoenzymes play distinct roles in TopoII-based chemotherapy, which points to the need of developing isoenzyme-specific drugs for cancer chemotherapy in order to reduce the risk of treatment-related secondary malignancies (Azarova et al., 2007[3]). This task can be helped by the recent resolution of the crystal structure of a large fragment of human TopoIIβ in a ternary complex with DNA and etoposide (Wu et al., 2011[80]). 

Another important therapeutic issue is the development of etoposide resistance. One player in this phenomenon is the double minute 2* (MDM2*) gene encoding an ubiquitin ligase involved in proteasome-mediated protein degradation. Interestingly, a single nucleotide polymorphism (SNP) SNP309 (T/G) in the gene promoter produces *MDM2* upregulation, and results in increased susceptibility to cancer and decreased response to radiation therapy and DNA-damaging drugs including TopoII poisons. Indeed cancer cell lines homozygous for SNP309 are selectively resistant (10-fold) to etoposide, mitoxantrone, amsacrine, and ellipticine. This effect arises from the ability of MDM2 protein to bind and target TopoII for degradation. Knockdown of MDM2 by RNAi stabilizes TopoII and decreases the resistance to TopoII-targeting drugs. Given the frequency of SNP309 in the general population (40 % of T/G heterozygosity and 12 % of G/G homozygosity), this polymorphism may represent a relatively common determinant of drug sensitivity with important implications for personalized cancer chemotherapy (Nayak et al., 2007[55]).

### Processing and repair of the protein-DNA covalent adducts 

The prolonged presence of TopoII-cleavage complexes and their associated single and double strand DNA breaks interfere with a number of DNA transactions, such as DNA replication and transcription, and must be efficiently removed by the cell. The first step in this process is the displacement of the protein cap from 5'-end of the break in order to make it accessible to DNA repair machineries. 

A few indirect mechanisms have been implicated in resolving the covalent link between TopoII and DNA. For instance, in the fission yeast *Schizosaccharomyces pombe* the nuclease activity of the Mre11/Rad50/ Nbs1 (MRN) complex, which is essential for repairing DSBs, removes TopoII from the DNA (Hartsuiker et al., 2009[27]). This process requires also the CtIP function, a BRCA1 interacting protein (Wong et al., 1998[79]; Yu et al., 1998[85]), which is recruited to sites of DNA damage (Sartori et al., 2007[67]). However, describing a new *Saccharomyces cerevisiae* mutant allele, Hamilton and Maizels observed that Mre11 have a role in response to topoisomerase poisons that can be also independent of its function in DSBs repair (Hamilton and Maizels, 2010[24]). These observations reveal a role of the MRN complex and CtIP in the cell resistance to a clinically important group of anticancer drugs. 

Another mechanism has been discovered by Zhang and colleagues (Zhang et al., 2006[87]) and involves the proteasome-mediated degradation pathways in the processing of TopoII-DNA adducts. Using a number of experimental systems, including TopoIIβ knock-out mouse embryonic fibroblasts, TopoIIβ small interfering RNA knock-down PC12 cells, as well as post-mitotic neurons in which TopoIIα is absent, the authors have proven that etoposide-induced DNA damage signals are attenuated upon proteasome inhibition with MG132. Moreover, by the analysis of the response to etoposide treatment in four different tumor cell lines, Fan and coworkers have demonstrated that the proteasome activity contributes to the processing of TopoIIcc into protein-free DNA breakage for subsequent sensing and repair of the damage in a transcription-dependent manner (Fan et al., 2008[16]). 

More recently, a human 5**'**-tyrosine phosphodiesterase has been identified for the excision of TopoII-DNA adducts (Cortes Ledesma et al., 2009[12]). TTRAP (TRAF and TNF receptor-associated protein) is a member of Mg^2+^/Mn^2+^-dependent family of phosphodiesterases that efficiently restore ligatable 5'-phosphate termini on DNA double-strand breaks. By analogy with the enzyme that removes TopoI-DNA adducts (tyrosine phosphodiesterase TDP1 (Nitiss et al., 2006[57])), it has been renamed tyrosine phosphodiesterase 2 (TDP2) and represents the major 5'-TDP activity in vertebrate cells (Zeng et al., 2011[86]; Gao et al., 2014[18]). Notably, cellular depletion of TDP2 results in increased sensitivity to etoposide-induced DSBs (Cortes Ledesma et al., 2009[12]) and raises the possibility that TTRAP/TDP2 plays a role in cancer development and response to therapeutic treatments. 

Processing of TopoIIcc leads to cytotoxic DSBs. A direct correlation has been demonstrated between the inability to repair DSBs and cell sensitivity to topoisomerase poisons (Hansen et al., 2003[26]). Proliferating cells use different pathways to repair DSBs depending on cell cycle phases (Kakarougkas and Jeggo, 2014[30]). In G1 DSBs are repaired by the low-fidelity non-homologous end-joining (NHEJ) pathway while homologous recombination (HR) plays a role in S and G2. Interestingly, knock out of DNA ligase IV (*LIG4^-/-^) *and of the Ku70 subunit of Ku heterodimer (*KU70^-/-^) *in chicken DT40 cells, which completely impairs NHEJ, makes the cells extremely sensitive to etoposide. In contrast, DNA repair and recombination protein *RAD54^-/-^* knock out cells (defective in HR) are much less hypersensitive. Collectively these results provide evidence that NHEJ is the predominant pathway for the repair of etoposide-induced TopoII-mediated DNA damage (Adachi et al., 2003[1]). In agreement with this conclusion, Malik and coworkers have found that the fission yeast orthologs of Ku70 (hdf1) and Ku80 (hdf2), and other genes required for NHEJ, are important for the survival of *Saccharomyces cerevisiae* following exposure to etoposide (Malik et al., 2006[44]). Moreover, Chen and co-workers (Chen et al., 2007[10]) have reported that the impairment of Ku70 function diminishes cellular capability to repair DSBs induced by etoposide and doxorubicin, thus enhancing cell-killing activity. Notably, Ku70 mutations that mimic acetylation of specific lysine residues suppress the ability of Ku70 to bind DNA and render prostate cancer cell more susceptible to the effect of etoposide suggesting a role of Ku70 acetylation in the effect of histone deacetylase (HDAC) inhibitors (Chen et al., 2007[10]). Finally, the analysis on several human tumor cells shows that NHEJ-mediated repair of etoposide-induced DNA lesions requires active transcription or replication of damaged DNA (Fan et al., 2008[17]), reinforcing the hypothesis that the interference with transcription and replication machineries has a major role in the etoposide cytotoxicity.

### DNA damage response

DNA damage induces an evolutionary conserved network of sensors, mediators and effectors, called DNA damage response (DDR), that sense the damage and spread the signal throughout the cell by a signal amplification cascade. As a consequence cell cycle progression is slowed-down or arrested to prevent the duplication and transmission of damaged DNA to daughter cells and to coordinate DNA repair pathways (Bartek and Lukas, 2007[4]). 

Single-stranded DNA and DSBs are sensed by specialized complexes that recruit to the lesions and activate two large protein kinases, ataxia telangiectasia and Rad3-related (ATR) and ataxia-telangiectasia mutated (ATM), resulting in the local phosphorylation of the histone variant H2AX (Maréchal and Zou, 2013[45]). Phosphorylated H2AX (called γH2AX) in turn recruits additional ATM complexes in a positive feedback loop, thereby increasing local ATM activity and causing the spread of γH2AX along the chromatin with the formation of the so-called γH2AX foci. 

Long stretches of single-stranded DNA derived by replication stress, are bound by the single-stranded DNA-binding protein replication protein A (RPA) and represent the signal for ATR recruitment. Therefore, while DSBs primarily activate ATM, processing of DSBs by specific endonucleases in S and G2 phases can produce extended single-stranded DNA regions, resulting in ATR activation indicating that both ATM and ATR kinases may be engaged at the same lesion. The local increase in the activity of these kinases causes the activation of two additional checkpoint kinases, Chk2 and Chk1 respectively, that diffuse throughout the nucleus and give raise to signaling pathways that converge on key decision-making factors, such as p53 and the cell-division cycle 25 (CDC25) phosphatases. DDR activation triggers a deep reorganization of nuclear functional compartments with the formation of discrete nuclear foci to which damaged DNA, DNA repair enzymes and auxiliary factors are recruited (Giglia-Mari et al., 2011[20]; Polo and Jackson, 2011[62]; Maréchal and Zou, 2013[45]). 

As expected from its ability to induce DSBs, etoposide triggers the activation of ATM and of its downstream kinase Chk2. Mutations of the ATM kinase, as in the case of ataxia telangiectasia (AT) patients, result in hypersensitivity to etoposide (Caporossi et al., 1993[8]). In particular, loss of the G2/M checkpoint in AT cells results in the execution of mitosis even in the presence of unrepaired etoposide-induced DNA breaks leading to chromosomal abnormalities (Nakada et al., 2006[54]). The activation of ATM kinase triggered by etoposide eventually leads to the formation of subnuclear repair foci containing the MRN (Mre11/Rad50/NBS1) complex and indistinguishable from ionizing radiation-induced foci (IRIF) (Maser et al., 1997[46]). The focal accumulation of DNA repair factors, including MRN complex and γH2AX is a key cytological signature of the DNA damage response. Although these foci have been extensively studied by light microscopy, little is known about their ultrastructure. Using correlative light microscopy and electron spectroscopic imaging (LM/eSI) Dellaire and colleagues (Dellaire et al., 2009[14]) have characterized the ultrastructure of chromatin and DNA repair foci within the nuclei of normal human fibroblasts in response to etoposide. They have observed a global decrease in chromatin density, which is accompanied by the formation of invaginations of the nuclear envelope. By time course experiments with dual immunogold labeling of Mre11 and γH2AX they have shown that DNA repair foci are highly dynamic and that late foci exhibit a highly organized chromatin arrangement distinct from earlier repair foci (Dellaire et al., 2009[14]). Interestingly, we have found that etoposide also exerts an effect on the functional organization of replication foci and factories, which are the subnuclear compartments where DNA replication takes place. During S phase, this drug progressively affects the distribution of replication proteins, triggering the dispersal of replication factories and inducing the formation of large nuclear foci containing single-strand DNA binding protein RPA (Rossi et al., 2006[64]). The subnuclear reorganization of replicative structures is also accompanied by a change in the phosphorylation pattern of replication proteins, i.e. RPA and DNA ligase I (Montecucco et al., 2001[49]; Rossi et al., 2002[65]) and requires the activity of ATR kinase and the cooperation of Nbs1, a partner of Mre11 nuclease in the MRN complex. Notably cells exposed to the topoisomerase II inhibitor, ICRF-193, or to the radiomimetic drug bleomycin, do not undergo the dispersal of replication factories even if checkpoint kinases are activated. Therefore, dispersal of replication foci elicited by etoposide does not depend on topoisomerase II inhibition or on the formation of DSBs *per se*, but requires the processing of the poisoned cleavage complexes. 

The effect on the subcellular distribution of proteins is not limited to the replication compartments. Indeed, using spatial proteomics, a mass spectrometry-based approach for measuring the subcellular distribution of the proteome, Lamond group has recently observed that etoposide treatment of HCT116 cells results in the dissociation of the proteasome from inhibitory proteins in the cytoplasm and its re-location in the nucleus with the ensuing association with proteasome activators (Boisvert et al., 2010[7]).

## Chromatin Perturbation and Transcription

Nuclear functions such as DNA replication and transcription are carried out within a highly organized three-dimensional environment, which starts with the packaging of DNA and associated proteins into chromatin. Post-translational histone modifications (PTMs) by affecting the higher-order chromatin structure directly influence the gene function, either activating or inhibiting transcription depending on the type of PTM, the residue which is modified and the distribution of modified histones along the gene (Schneider and Grosschedl, 2007[68]). Among histone PTMs, acetylation appears to influence the response to etoposide treatments. 

The acetylation state of lysine residues, including those at the N-terminal 'tail' of the histones, is controlled by histone deacetylases (HDACs) and histone acetyltransferases (HATs) that also modulate the acetylation/deacetylation status of non-histone proteins, including transcription factors and co-regulators (Hermanson et al., 2002[28]; Glozak et al., 2005[21]; Liu et al., 2009[40]). Several experimental evidences link HDACs and HATs to TopoII and to the response to etoposide treatment. For instance, HDAC1/2 have been found in a functional complex with TopoIIα/β *in vivo* (Tsai et al., 2000[74]), and TopoII is an integral component of the NuRD complex which also contains HDAC1/2 and an ATP-dependent chromatin remodeling activity (Tong et al., 1998[73]). These interactions may account for the observation that the etoposide concentration required to inhibit cell growth by 50 % (IC_50_) is significantly decreased when cells are subjected to pre-treatment with the HDACs inhibitor (HDACi) trichostatin A (TSA). Along this line it is worth noticing that HDACi also induce sensitization to etoposide of multidrug-resistant cancer cells (Hajji et al., 2008[23]; Hajji et al., 2010[22]). This effect is associated with an increased acetylation of particular lysines on histone H3 and H4, including lysine 16 on histone H4 (H4K16). Notably, overexpression of histone acetylase hMOF, known to target H4K16, can mimic HDACi treatment, while overexpression of SIRT1 deacetylase counteracted the effect, pointing to hMOF and SIRT1 activities as critical parameters in HDACi-mediated sensitization to etoposide. Moreover, TSA treatment displaces TopoIIβ from its binding to heterochromatin (Cowell et al., 2011[13]). Although the underlying molecular mechanism is till to be defined, the displacement can convert TopoIIβ to an effective relevant target for topoisomerase poisons. 

The melanoma antigen 1 (MageA1) is one of the first identified tumor-specific antigens (van der Bruggen et al., 1991[75]). Emerging data suggest the potential involvement of MAGE family proteins in modulating cell survival with two MAGE-II members, Necdin and hNRAGE, oppositely modulating p53 functions (Taniura et al., 1999[71]; Wen et al., 2004[77]). Claudio Schneider group (Monte et al., 2006[48]) has reported that MageA2 interacts and represses p53 activity by recruiting transcription repressors HDACs to p53 transcription sites and inducing histone hypoacetylation in melanoma cells. Interestingly, melanoma cells expressing MAGE-A genes are refractory to etoposide-induced apoptosis. The correlation between MAGE-A expression and resistance to apoptosis has been validated in melanoma cell lines, where combined TSA and etoposide treatment restores the p53 response and reverts the chemoresistance of cells expressing high levels of MAGE-A.

Chromatin organization and chromosome territories may be actually targets of etoposide treatment. In this regard it is worth noticing that repositioning of TopoIIcc could account for the relocation of the ETO/MTG8/ RUNX1T1 gene (located on chromosome 8) in physical proximity to the AML1 gene (on chromosome 21) observed in cycling cells upon etoposide treatment. Rubtsov and colleagues (Rubtsov et al., 2008[66]) have suggested a link between this redistribution and the t(8;21)(q22;q22) translocation that is frequently observed in acute myeloid leukemia (AML) (Zhang et al., 2002[89]). 

Another connection between etoposide, chromatin organization and gene expression has been recently reported by Huang and coworkers (Huang et al., 2012[29]). These authors showed the ability of etoposide and other topoisomerase inhibitors to un-silence gene expression by repressing transcription of *cis* acting non-coding RNA. This fascinating finding concerns the dormant allele of *UBE3A* gene whose mutation causes the neurodevelopmental disorder known as Angelman syndrome. In neuron, the paternal allele of the ubiquitin protein ligase E3A (*UBE3A*) is intact but epigenetically silenced by an imprinted antisense RNA. Etoposide treatment un-silences the paternal allele reducing the transcription level of the *cis* acting antisense RNA. This observation, besides suggesting a potential therapy for patient bearing mutations in the *UBE3A* maternal allele, sheds a new light on additional molecular mechanisms and therapeutic potential of TopoII poisons.

A further mechanism through which etoposide can down-regulate transcription of specific genes, has been unveiled by Takami and colleagues (Takami et al., 2011[70]) that, by screening a library of phage-displayed peptides, identified transcription factor E2F-4 as an etoposide binding protein. Binding of E2F-4 to etoposide inhibits gene transcription mediated by the heterodimeric E2F-4/DP complexes in the nucleus. 

Etoposide can affect transcription also through the bridging integrator-1 (BIN1) protein originally identified as a c-MYC-interacting pro-apoptotic tumor suppressor. Several splice variants of BIN1 harbor a MYC-binding domain (MBD), thus retaining the ability to physically interact with and hence to inhibit the oncogenic functions of c-MYC. Krainer group (Karni et al., 2007[32]) showed that etoposide enhances the interaction of E2F1 transcription factor with the BIN1 gene promoter. Notably, suppression of BIN1 expression using an antisense (AS) technique attenuated the cell death mediated by E2F1 and etoposide.

Etoposide can also affect gene expression by modulating alternative splicing profiles. Alternative splicing is a co-transcriptional event that expands the coding capacity of the genome by affecting the vast majority (more than 90 %) of human genes (Pan et al., 2008[58]). A proteomic analysis to quantify DNA damage-regulated changes in phosphoproteome, acetylome, and proteome in human osteosarcoma cells treated with etoposide showed that a significant fraction of the hits corresponds to proteins involved in RNA metabolism such as THRAP3 (thyroid hormone receptor-associated protein 3), which is part of a multiprotein complex that controls Cyclin D1 mRNA stability, and the splicing-regulator phosphatase protein phosphatase Mg^2+^/Mn^2+^ dependent 1G (PPM1G), a nuclear member of the PP2C family of Ser/Thr phosphatases (Beli et al., 2012[5]). In particular, the splicing-regulator phosphatase PPM1G is recruited to sites of DNA damage, while the splicing-associated protein THRAP3 is excluded from these regions.

In line with the results of large-scale proteomic analysis (Matsuoka et al., 2007[47]; Beli et al., 2012[5]) we have recently observed that the cell response to DNA damage affects the level and post-translational modifications of RNA binding proteins including splicing regulators belonging to the SR family and hnRNP proteins (Leva et al., 2012[38]; Montecucco and Biamonti, 2013[50]). Exposure of the cells to 100 µm etoposide for 3 h induces dephosphorylation of splicing factor SRSF1 (previously known as SF2/ASF) that controls alternative splicing of several genes involved in the choice between pro- and anti-apoptotic pathways (Moore et al., 2010[51]) including Ron oncogene (Biamonti et al., 2014[6]; Ghigna et al., 2005[19]) and tumor suppressor BIN1 (Karni et al., 2007[32]). Changes in the phosphorylation status of SRSF1 in response to DNA damage results in modulation of the alternative splicing profile of caspase 9 (Leva et al., 2012[38]; Shultz et al., 2010[69]) pointing to alternative splicing as a molecular mechanism actively involved in the apoptotic effect of this drug. The list of genes whose alternative splicing is affected by genotoxic stress includes the CDC25 phosphatase that is essential for cell cycle control under normal conditions and in response to DNA damage. Notably, etoposide, doxorubicin, and the TopoI poison camptothecin alter the balance between CDC25 splice variants in human breast cancer cell lines both at the mRNA and protein levels (Albert et al., 2012[2]).

## Cell Death

### Apoptosis

Failure to properly repair DNA damages induces the activation of the process of programmed cell death or apoptosis that involves a family of conserved cysteine-dependent proteases called caspases (Riedl and Shi, 2004[63]).

**Figure 2:**
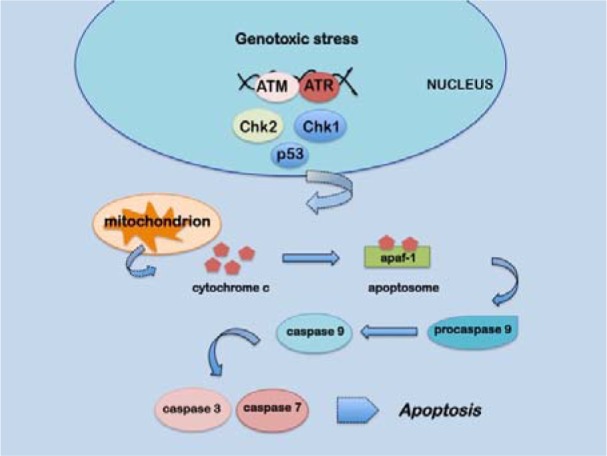
Schematic representation of the apoptotic pathway induced by genotoxic stress described in the text

It is known that high etoposide concentrations can trigger caspase-mediated apoptosis which mainly occurs through the cytocrome c/caspase 9 pathway (Wang et al., 2006[76]). After a genotoxic insult, in the early phases of apoptosis, cytocrome c is released from the mitochondria into the cytoplasm and binds to apoptotic protease activating factor 1 (apaf1) forming the apoptosome that, in turn, binds and cleaves procaspase 9 leading to its activation. Caspase 9 is then able to activate other effector caspases such as caspase 7 and caspase 3 triggering further apoptotic processes (Figure 2(Fig. 2)). 

There are also evidences of the involvement of a Fas ligand (FasL) pathway in etoposide-mediated apoptosis (Kaufmann and Earnshaw, 2000[34]). Etoposide treatment triggers FasL binding to its receptor (FasR) on cell membrane. This results in trimerization of the FasL-FasR complex and leads to the formation of the so-called death-inducing signalling complex (DISC). DISC-binding protein FADD is likely to bind procaspase 8 and to promote its activation to capsase 8 through self-cleavage. Caspase 8, in turn, interacts with some effector caspases such as caspase 3.

As stated above etoposide treatment induces the formation of DSBs also in non-replicating cells thus perturbing gene transcription. This has been proven in resting human T lymphocytes where etoposide treatment activates DDR and induces phosphorylation of ATM and of its substrates, H2AX and p53. The final effect is the activation of the pro-apoptotic PUMA (p53 upregulated modulator of apoptosis) protein and of caspases, leading to apoptotic cell death. Intriguingly, in resting cells inhibition of ATM activity with Ku55933 blocks DDR and apoptosis by preventing PUMA expression and caspase activation, while the same protocol increases the cytotoxic effects of etoposide on proliferating tumor cells (Korwek et al., 2012[36]). This opens new avenue for antitumor strategies. 

The tumor suppressor p53 plays an important role in etoposide-induced apoptosis and is finely regulated by the drug (Dey et al., 2010[15]). It has been recently shown that the Nemo-like serine/threonine kinase (NLK), a member of the mitogen activated kinase protein family, is required for p53 activation after etoposide-induced DNA damage (Zhang et al., 2014[88]). The expression of the NLK gene is upregulated in response to DDR pathways, even if the mechanism involved is still unclear. The interaction with NLK stabilizes p53 through the inhibition of the MDM2-mediated degradation of the protein, mediated by the ubiquitin-proteasome system (Zhang et al., 2014[88]; Kruse and Gu, 2009[37]). NLK may also increase the sensitivity to etoposide-induced apoptosis by suppressing transcriptional factors such as NF-κB (Yasuda et al., 2004[83]; Fan et al., 2008[17]). 

### Autophagy

**Figure 3:**
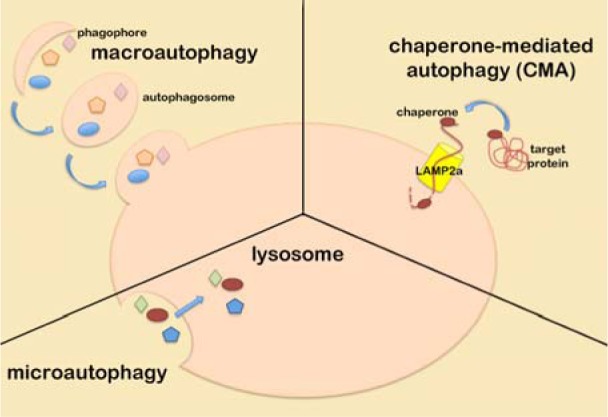
Schematic representation of the autophagic pathways described in the text

Autophagy is a catabolic pathway that helps cells to degrade unnecessary or misfolded components and to recycle nutrients during metabolic stress. Although autophagy can be seen as a cellular pro-survival mechanism, it can also promote non-apoptotic programmed cell death (reviewed by (Nagelkerke et al., 2015[53][52]). The autophagic pathways can be divided into macroautophagy, microautophagy, and chaperone-mediated autophagy (Figure 3(Fig. 3)). 

In macroautophagy, a membranous structure called autophagosome is formed through different steps: initiation, that requires the inhibition of mTOR pathway (Kamada et al., 2000[31]); nucleation*, *that is controlled by the activity of hVps34 kinase and its interacting proteins beclin-1 and p150/Vps35 to form the so called PI-3K (phosphatidylinositide 3-kinase) complex on the membrane of the preautophagosome; enlongation*,* in which the preautophagosome membrane grows and fuses its extremities thanks to the action of the atg5-atg12-atg16 protein complex. Once the autophagosome is correctly assembled it maturates by fusing with a lysosome giving raise to an autophago-lysosome (Liang et al., 2008[39]). Macroautophagy can also be a selective pathway since the component of autophagosome, LC3 protein, interacts with P62, leading polyubiquitinated proteins into the autophago-lysosome for degradation (Pankiv et al., 2007[59]). 

Microautophagy is a process involved in the degradation of entire regions of cytosol directly performed by lysosomes through membrane invagination or evagination and without the formation of autophagolysosomes (Klionsky, 2005[35]). Finally, chaperone-mediated autophagy is a selective mechanism by which hsc70 chaperone bound to KFERQ-motif-containing proteins, binds to the lysosomial membrane by interacting with LAMP2a receptor and internalizes the unfolded target protein into the lysosome for degradation. 

Etoposide is a well-known trigger of apoptotic pathways but recent findings suggest its involvement in autophagic pathways as well. It is still unclear, however, if the induction of autophagic processes by etoposide causes cell death or plays a pro-survival function. In this regard it is worth noticing that Katayama and colleagues have shown that etoposide induces an autophagy-dependent production of ATP in multiple glioma cells that actually protects cells and may contribute to drug resistance (Katayama et al., 2007[33]). This increase in ATP levels can not be blocked by glucose starvation, but is prevented by preincubation with the autophagy inhibitor 3-methyladenine (3-MA), or by siRNA-mediated down-regulation of beclin 1, or by the mitochondrial inhibitor oligomycin. All these treatments, by inhibiting the production of autophagy-induced ATP, increase non-apoptotic cell death. These results have been confirmed by Bu Sham and colleagues in HepG2 cells (Xie et al., 2011[81]). Collectively these analyses support the idea that DNA damaging agents such as etoposide may induce an autophagy-associated ATP surge that protects the cells and contributes to drug resistance (Katayama et al., 2007[33]). 

However, a study aimed at dissecting the mechanism by which etoposide induces cell death, showed that etoposide treatment of Hep3B hepatoma cells causes not only apoptotic but also autophagic phenotypes. Autophagy inhibition by 3-MA and caspase inhibition by zVAD-fmk effectively decreased autophagic and apoptotic phenotypes, respectively. However, both drugs when used separately only partially prevented cell death indicating that etoposide concomitantly induces autophagic cell death and apoptosis in Hep3B cells (Yoo et al., 2012[84]). 

## Acknowledgements

We apologize to colleagues whose work could only be cited indirectly. This work has been supported by grants from the Associazione Italiana per la Ricerca sul Cancro AIRC, the PNR-CNR Aging Program 2012-2014 CNR-MIUR, the Flagship project Epigen CNR-MIUR, the MbMM project 2014-2015 Accordo Quadro Regione Lombardia-CNR to GB. FZ is a student of the PhD program in Genetics, Molecular and Cellular Biology of the University of Pavia and is supported by a fellowship of the MbMM project.
